# Losing control without your smartphone: Anxiety affects the dynamic choice process of impulsive decision-making and purchase

**DOI:** 10.3389/fnins.2023.998017

**Published:** 2023-03-16

**Authors:** Dan-Yang Gui, Yu Dai, Zhichao Zheng, Shixiong Liu

**Affiliations:** Department of Marketing, College of Management, Shenzhen University, Shenzhen, China

**Keywords:** intertemporal choice, anxiety, DDM, smartphone addiction, consumer behaviors

## Abstract

Different interacting contexts influence the decision-making process, as revealed by the computational modeling. Through four studies, we investigated how smartphone addiction and anxiety influenced impulsive behaviors, along with the underlying psychological mechanisms and dynamic decision-making processes. In the first and second studies, we found no significant correlation between smartphone addiction and impulsive behavior. However, in the third study, we found that smartphone separation increased impulsive decision-making and purchases, and state anxiety, but not trait anxiety, mediated this effect. We explored the dynamic decision-making process using a multi-attribute drift diffusion model (DDM). The results showed that anxiety triggered by smartphone separation changed the trade-offs between decision weights for the fundamental components of the dynamic choice process. In the fourth study, we investigated why smartphone addiction led to increased anxiety and found that extended-self was a mediating factor. Our findings show that smartphone addiction was not correlated with impulsive behaviors, but was correlated with state anxiety in the context of smartphone separation. Further, this study shows how emotional states triggered by different interacting contexts affect the dynamic decision-making process and consumer behaviors.

## 1. Introduction

Impulsive decision-making and purchases are typical impulsive behaviors. According to the traditional delay discounting model (TDM), individuals with more patience have lower discounting rates (Frederick et al., 2002). Recently, researchers have focused on the dynamic decision-making processes. The drift diffusion model (DDM) is a good dynamic model to explain value-based decisions (Thomas et al., 2019; Zhao et al., 2019). Additionally, smartphones have become an integral part of life and have been referred to as an extension of the self (Belk, 2013). When users are separated from their smartphones, their emotions may be adversely affected. Smartphone separation may lead to anxiety, which is related to social problems such as the Fear of Missing Out (FoMO) (Przybylski et al., 2013) and social threats (Tams et al., 2018). It may also cause strong feelings of distress (Yildirim and Correia, 2015), and have various negative consequences for personal decision-making and wellbeing. Thus, we predicted that the diversity of decision contexts, such as having or not having smartphones, may impact an individual’s emotional state and behavioral consequences. In addition, different contexts may influence the decision-making process, as revealed by the computational model.

### 1.1. Theoretical framework

#### 1.1.1. Impulsive decision-making and purchases

Daily life presents several opportunities for impulsive behaviors, such as making unnecessary purchases at the supermarket, neglecting studying, or working to participate in recreational activities. In the field of psychology, researchers consider impulsivity to be a tendency to act prematurely without foresight. This finding is thought to be significantly related to personality traits. High levels of impulsivity often correspond to a tendency to engage in unplanned activities (Dalley et al., 2011).

This study explored impulsive behaviors related to intertemporal choices and impulsive purchases. Intertemporal choice is a paradigm of impulsive decision-making that describes how individuals balance the costs and benefits at different time points to make various judgments and decisions (Frederick et al., 2002). An impulsive purchase is a sudden, compelling, and hedonically complex purchasing behavior that precludes thoughtful and deliberate consideration of all information options and consequences (Kacen and Lee, 2002). In such cases, the individual focuses on achieving immediate gratification (Sengupta and Zhou, 2007). People with addiction often engage excessively in activities offering immediate gratification, regardless of the potential negative consequences (Stacy and Wiers, 2010; Cheng et al., 2021). Impulsive purchases are accompanied by more intense emotions than deliberative purchases, and are characterized by a lack of forethought and excessive buying (Dawson and Kim, 2013).

#### 1.1.2. Smartphone addiction and anxiety

Smartphone addiction is a behavioral addiction characterized by cognitive impairment, loss of control, mood changes, withdrawal, interpersonal conflict, and recrudescence (Lee et al., 2014). Separating from smartphones may cause strong perceptions of distress (Yildirim and Correia, 2015), and in turn, have various negative consequences for wellbeing and healthcare problems.

Smartphone addiction is influenced by several factors, such as interpersonal communication and social support. Individuals with a stronger motivation for interpersonal communication tend to use smartphones more frequently (Cho and Lee, 2017; Wilmer et al., 2017). Furthermore, many studies have suggested that impulsivity is significantly associated with addictive behavior (Moffitt et al., 2011; Ersche et al., 2012, 2013). According to the dual-system theory of addiction, addictive behavior is caused by an imbalance between the reflective and impulsive systems of the brain (Stacy and Wiers, 2010). People with substance addiction, such as drug or alcohol addiction, consistently exhibit high impulsivity (Mitchell et al., 2005; Ersche et al., 2012). Impulsivity also plays a critical role in non-material addiction, and individuals with pathological gambling or Internet addiction are more likely to make impulsive choices (Alessi and Petry, 2003).

Furthermore, previous studies on intertemporal choice have employed a delay-discounting task to indicate or predict the degree of addiction in people with nicotine and alcohol addiction. The findings showed that these individuals exhibited a higher discount level for a substantial, delayed reward than the control group (Monterosso et al., 2007; Bradford, 2010). Therefore, we hypothesized that individuals with a high degree of smartphone addiction would be more inclined to engage in impulsive decision-making when confronted with intertemporal choices. That is, we predicted that individuals with smartphone addiction would be more likely to make impulsive decisions during their daily purchasing behavior.

Smartphone addiction has negative effects on mental health and wellbeing (Samaha and Hawi, 2016), and Kim et al. (2015) found that individuals who excessively use smartphones tend to have severe depression and anxiety, causing social and emotional dysfunction. Hartanto and Yang (2016) also discovered an association between smartphone separation and symptoms of severe anxiety. Smartphone separation is defined as being without a smartphone, or disconnected from a smartphone (Hartanto and Yang, 2016; Ward et al., 2017). Anxiety caused by smartphone separation may influence impulsive decision-making and purchases. Anxiety is defined as the complex emotional state of tension, uneasiness, worry, annoyance, and feelings of being threatened or trapped in an unpleasant situation (Spielberger et al., 1983). It can be divided into state anxiety and trait anxiety. State anxiety is a specific, temporary emotional state produced by external stimuli. Trait anxiety is an aspect of an individual’s personality and is relatively stable (Endler and Kocovski, 2001). Therefore, we propose that smartphone separation affects individuals’ state anxiety and influences impulsive behaviors. Additionally, we predicted that smartphone addiction would play an important role in the effects of smartphone separation.

#### 1.1.3. Intertemporal choice, discounting rate, and DDM

Intertemporal choice is a classic paradigm that measures an individual’s impulsive behavior (Rung and Madden, 2018). According to theories of delay discounting, individuals prefer immediate rewards. Thus, delay causes a sharp devaluation of future outcomes. Individuals with more patience tend to choose larger and later rewards (LL), whereas those with less patience choose smaller and sooner rewards (SS) (Kim et al., 2012). The discounting rate (*k*) describes the patience of individuals, with a lower *k* value representing higher patience (Green and Myerson, 2004; Odum, 2013; Rung and Madden, 2018). However, intertemporal choice is not instantaneous, but involves a complex dynamic decision-making process. Thus, we propose that the TDM does not adequately explain the trade-off between reward and delay, or the dynamic underlying mechanism of intertemporal choice. Furthermore, *k* in TDM indicates how the subjective value of reward discounts with time delay (Frederick et al., 2002), which demonstrates the result of intertemporal choice; for example, we could know someone is impulsive through TDM, and *k* means how she or he was impulsive. The pros of TDM include that it is simple and clear for decision-making or scenarios which do not need to know the underlying processing. The con is that it cannot reveal the underlying processing.

On the other hand, the DDM, used in intertemporal choice, can explain why someone is impulsive. DDM demonstrates the multi-attribute dynamic cognitive processes during intertemporal choice, in which decision-makers evaluate different attributes (time delay and money) of decision-making, and form a trade-off between attributes that contribute to individual *k*, which mean*s* individuals have varied *k* due to different perceptions and evaluations of attributes (Krajbich and Rangel, 2011; Ratcliff et al., 2016; Thomas et al., 2019). The cons are that DDM is more complex and has more parameters than TDM; the pros including that DDM can provide more information and underlying attribute-evaluation processes, through which we know how to influence people’s perception and choices.

#### 1.1.4. DDM and addiction

Drift diffusion model is a computational model for two-alternative and multi-option forced choices. It provides a dynamic modeling approach to explain and predict decision-making outcomes based on psychological and computational cognitive aspects (Ratcliff and McKoon, 2008; Wiecki et al., 2013). The DDM assumes that relative evidence accumulates over time (Ratcliff and McKoon, 2008). Decisions are generated by a noisy process that accumulates relative evidence (*R*) that one option is better in comparison to the other. The relative evidence (*R*) follows a diffusion process and evolves in small time increments according to a stochastic difference equation, *R_*t*_*_+ 1_ = *R*_*t*_ + *v* + *S*_*t*_. Here, *v* is the drift rate, and *S* represents the mean-zero Gaussian noise. Markov chain Monte Carlo sampling methods were used to estimate the joint posterior distribution of all model parameters.

Specifically, DDM models the decision process into a two-alternative option task, and relative evidence for one option over the other option is sampled and accumulated during the decision stage. Each option is represented by an upper boundary and a lower boundary. The accumulated evidence for each option is compared with that of the other options, and the decision is made when the relative evidence for any option exceeds the boundary. The speed at which evidence is accumulated is referred to as the drift rate. The drift rate corresponds to the relative preference in the evaluation process for one reward over the other. Owing to noise during the drift process, the reaction time across the boundary and selected options vary in different trials (Zhao et al., 2019).

There have been several studies on addiction using DDM, but the results are complex. One study on rodents found that excessive drinking in rats showed an inability to delay reward, which increased the risk of alcohol use disorder (Linsenbardt et al., 2017). Nonsmokers had a higher drift rate than smokers in the condition where negative words were associated with smoking (Gao et al., 2022). The DDM model suggests that the lower prosociality of individuals with methamphetamine use disorder could be attributed to the lower weight placed on others’ benefits (Li et al., 2022). However, in other studies, heroin-addicted mothers showed no significant group differences in any parameter of the examined DDM compared to the healthy control group (Eikemo et al., 2019). There were also no robust group differences in DDM parameters between heavy drinking and drinking in moderation (Copeland et al., 2022).

Previous studies have adopted DDM to explore the dynamic decision-making process and have indicated that DDM is a good illustration of value/preference-based choice (Basten et al., 2010; Westbrook et al., 2020). It is being increasingly employed in the fields of computational psychology and neuroscience. Furthermore, DDM is considered to capture multi-attribute dynamic accumulation during decision-making compared with traditional models (Forstmann et al., 2016). However, few studies have focused on the effect of personal characteristics or individuals’ emotional states on dynamic decision-making processes. Thus, we aimed to explore the relationship between individuals’ emotional states and dynamic decision-making processes.

Based on the above literature review, we employed DDM to explore the dynamic decision-making process of intertemporal choice in the context of smartphone separation. We predicted that individuals in the smartphone separation condition would be more likely to make impulsive decisions, which is reflected by the changed decision weights for reward or delay.

#### 1.1.5. Underlying psychological mechanisms of smartphone addiction and anxiety

From the above discussion, we predicted that smartphone addiction would increase state anxiety in the case of smartphone separation. However, it remains unclear as to why smartphone addiction increases anxiety. Previous studies have proposed several theories to explain the effects of smartphone separation and addiction on state anxiety. The first is the FoMO, the feeling of unease or total exhaustion resulting from missing out on peer activities or favorable alternatives (Przybylski et al., 2013). Previous research has demonstrated that FoMO has a negative effect on mental health indicators, such as life satisfaction and wellbeing (Przybylski et al., 2013). In smartphone separation, FoMO leads to higher state anxiety, because engagement with social media attracts people prone to FoMO. When the social demands of such individuals remain unfulfilled, they experience anxiety (Przybylski et al., 2013).

The second theory is social threat. Individuals with social phobia are more likely to perceive social threats in the environment than others (Amir et al., 2003). Furthermore, psychologists have discovered a new condition related to smartphone reliance known as nomophobia. Nomophobia is a disorder that develops because of smartphone separation and refers to the morbid fear, anxiety, and discomfort associated with technological disengagement (King et al., 2013). In the case of nomophobia, social threat is presented as an inability to engage with technologies such as e-mail, instant messaging, IP voice messaging, Twitter, and Facebook posts (King et al., 2014). Moreover, social threats have been implicated as the underlying mechanism of nomophobia and stress (Tams et al., 2018). Thus, we predicted that social threats can explain the effect of smartphone addiction on anxiety.

Mobile technology is an extension of the self, and has become an intimate component of personal entity. This notion is related to the theory of extended-self (Tian and Belk, 2005), which posits that people feel a strong sense of possession over certain objects that are infused with thoughts, memories, and social emotions. These include souvenirs, photographs, and letters, and individuals regard these items as a part of themselves. Smartphones provide people with a link to the past and serve as tools for transferring memories. Accidental loss of possessions, including material or digital belongings, is often experienced as a loss of the self (Belk, 2013). Consequently, when users are separated from their smartphones, they may feel a loss of self, which increases their state of anxiety.

Based on the theoretical framework, we propose the following hypotheses:

**H1:** Smartphone addiction is positively correlated with impulsive decision-making and purchases.

**H2:** Participants in the smartphone separation condition demonstrate higher impulsivity during decision-making and purchases than those in the control condition.

**H3:** State anxiety mediates the effect of smartphone separation on impulsive decision-making and purchases.

**H4a:** Smartphone separation changes the dynamic process of impulsive decision-making, and DDM measures this change, representing the decreasing decision weight on reward.

**H4b:** Smartphone separation changes the dynamic process of impulsive decision-making, and DDM measures this change, representing the increasing decision weight on delay.

**H5a:** FoMO mediates the effect of smartphone addiction on anxiety.

**H5b:** Social threat mediates the effect of smartphone addiction on anxiety.

**H5c:** Extended-self domain on smartphones mediates the effect of smartphone addiction on anxiety.

Therefore, our research aimed to explore (1) whether smartphone addiction is related to impulsive behaviors, (2) how trait or state anxiety affects the dynamic decision-making process, and (3) the underlying psychological and cognitive mechanisms of anxiety on impulsive behaviors.

## 2. Study 1

The aim of study 1 was to test hypothesis 1.

### 2.1. Method

In total, 68 healthy volunteers participated in this study (37 females and 31 males), with ages ranging from 19 to 23 years (the mean was 20.04 years). The sample included students from a local university and technical staff from the local community. We conducted the study on a local campus and community for 5 days, and the data were collected by a research assistant. We did not do simple-size estimation because we did not realize we should, this is a shortage for study 1, and we did sample-size estimation in all next studies. All participants were right-handed, had normal vision (with or without correction), reported no history of affective disorders or neurological diseases, and were not taking any chronic medications. All participants provided written informed consent prior to the experiment.

We adopted a paper-and-pencil questionnaire to measure all of the variables. Participants completed the Smartphone Addiction Scale (SAS) (Kwon et al., 2013) which was designed to measure the degree of smartphone addiction. Responses were rated on a 5-point Likert-scale. Next, we used intertemporal choice (Frederick et al., 2002) to evaluate the impulsivity of participants’ decisions. Participants were required to decide whether to immediately receive *X* yuan or 200 yuan 2 months later (*X* = 10, 20,…, 200). We identified a point of indifference for each participant, that is, individuals with higher impulsivity would prefer immediate rewards (Zhang and Smith, 2018).

After completing the intertemporal choice task, the participants indicated their willingness to make impulsive purchases. Specifically, participants were asked to imagine the following scenarios based on a previous study (Gui et al., 2021), and the accompanying questions were considered a measure of impulsive purchase. (1) Restaurant scenario: “You planned to eat at your favorite restaurant but were informed that you must queue upon arrival. How long are you willing to wait before leaving?” The answers ranged from 5 to 60 min. (2) Movie scenario: “A new movie is released before the deadline for an important work report or examination. You have been looking forward to watching this movie for a long time. When will you choose to watch the movie?” Answers were measured on a 100-point scale, anchored by 1 = “before the deadline” and 100 = “after the deadline.” (3) Gift compensation scenario: “You won a ¥50 gift card in a lucky draw, but the gift delivery was delayed by 10 days because of a stock shortage. The organizer will provide an extra compensatory gift. How much do you hope that the compensatory gift is worth?” Responses ranged from 1 to 50. Finally, the participants provided their demographic information.

### 2.2. Data analyses

All statistical analyses were performed using SPSS (version 24.0; SPSS, Inc., Chicago, IL, USA), and the significance level was set at 0.05. A Pearson’s correlation was used to analyze the correlation between the degree of smartphone addiction and intertemporal choice.

### 2.3. Results

The results indicated no significant correlation between the degree of smartphone addiction and intertemporal choice (*p* > 0.05) among the three impulsive purchase scenarios (*p* > 0.05). The Cronbach’s alpha of SAS in study 1 was 0.883. This suggests that smartphone addiction did not correlate with impulsive decision-making or impulsive purchasing in the absence of specific conditions. Thus, hypothesis 1 was rejected.

### 2.4. Discussion

Study 1 revealed no significant correlation between smartphone addiction and impulsive decision-making or impulsive purchasing. Due to the limitations of paper-and-pencil questionnaires, we included only 68 participants. Thus, sampling issues may have resulted in inaccurate results that did not match our assumptions.

## 3. Study 2

Due to the small sample size in study 1, we expanded the sample size and changed the data collection methods in study 2 to re-test hypothesis 1. In study 2, we explored the relationship between smartphone addiction and impulsive behavior. By using larger samples and different methods, we wanted to make our conclusion more robust.

### 3.1. Method

A total of 185 healthy volunteers participated in this study (111 females and 74 males), with ages ranging from 28 to 40 years (the mean age was 24.85). We conducted the study online for 3 days. The sample size was estimated using G*Power 3 (Faul et al., 2007), in which the statistical power was 0.9 for the correlation and the one-way analysis of variance. The sample included students, grassroots and technical staff, junior and senior managers, and other professionals. All participants provided written informed consent before participating in the experiment.

We adopted WJX^1^ as a paperless online questionnaire platform. Online surveys are usually subject to concerns, such as an insufficient amount of time spent answering questions and multiple questionnaires being completed by the same participant. The survey platform navigated these issues by setting a minimum time limit required to complete the questionnaire and by preventing users with the same IP address or device from participating multiple times.

This procedure was similar to that used in study 1. Participants were asked to complete questionnaires designed to measure smartphone addiction (Kwon et al., 2013) and impulsive decision making (intertemporal choice) (Frederick et al., 2002). We measured impulsive purchasing behavior using the same three consumption scenarios (Gui et al., 2021) used in study 1. Finally, the participants provided their demographic information.

### 3.2. Data analyses

All statistical analyses were performed using SPSS (version 24.0; SPSS, Inc., Chicago, IL, USA), and the significance level was set at 0.05. We used Pearson’s correlation and a one-way analysis of variance to analyze the relationship between smartphone addiction and impulsive decision-making or impulsive purchases.

### 3.3. Results

We found no significant correlation between smartphone addiction and impulsive decision-making (*p* > 0.05), or among the three impulsive purchase scenarios (*p* > 0.05). A Cronbach’s alpha of SAS in study 2 was 0.812. Moreover, participants were divided into two groups, namely high and low smartphone addiction, using a median score (*M* = 33). A one-way analysis of variance indicated that there was no significant difference in intertemporal choice (*M*_*high–addiction*_ = 134.23, SD = 29.26; *M*_*low–addiction*_ = 135.57, SD = 25.86; *p* > 0.05, *F* = 0.092, η^2^ = 0.001) between the high-addiction and low-addiction groups. No significant relationship was observed for the restaurant scenario (*M*_high–addiction_ = 23.85, SD = 11.19; *M*_low–addiction_ = 23.67, SD = 11.29, *p* > 0.05, *F* = 0.010, η^2^ = 0.000), movie scenario (*M*_*high–addiction*_ = 71.12, SD = 27.53; *M*_low–addiction_ = 70.15, SD = 28.25; *p* > 0.05, *F* = 0.047, η^2^ = 0.000), and gift compensation scenario (*M*_high–addiction_ = 27.00, SD = 12.98; *M*_low–addiction_ = 25.58, SD = 13.89; *p* > 0.05, *F* = 0.436, η^2^ = 0.003). Thus, smartphone addiction was not significantly correlated with impulsive decision-making or impulsive purchases.

### 3.4. Discussion

Study 2 repeated the results of study 1, revealing that smartphone addiction was not correlated with impulsive behaviors in the absence of specific conditions. These results were inconsistent with hypothesis 1, which we attribute to our disregard for smartphone separation. Previous research has shown that smartphone separation triggers severe anxiety, which affects executive function (Hartanto and Yang, 2016). Therefore, we predicted that smartphone separation would increase state anxiety, thus affecting impulsive decision-making and purchase intentions.

## 4. Study 3

Study 3 aimed to examine hypotheses 2, 3, and 4. This study aimed to assess the effect of smartphone addiction separation on impulsive decision-making and impulsive purchase intention. We conducted a manipulated lab experiment using a 2 (smartphone separation: separated versus control) × 2 (smartphone addiction: high versus low) mixed design. Smartphone separation was a between-subjects factor, and smartphone addiction was a within-subjects factor.

### 4.1. Method

A total of 72 healthy volunteers (31 females and 41 males) participated in this study. Their ages ranged from 23 to 28 years (the mean age was 25.8 years). The sample size was estimated using G*Power 3 (Faul et al., 2007), in which the statistical power was 0.9 for the correlation and the one-way analysis of variance. The sample was comprised of local students and working professionals in Shenzhen. We conducted the experiment in the laboratory of the university for 2 weeks. All participants were right-handed, had normal vision (with or without correction), reported no history of affective disorders or neurological diseases, and did not take any chronic medications. All participants provided written informed consent before participating in the study.

The participants were randomly assigned to the control or smartphone separation groups. According to previous studies (Hartanto and Yang, 2016; Ward et al., 2017), the control group participants brought their smartphones into the laboratory, while participants in the smartphone separation group were asked to leave their smartphones in another room. All participants were instructed to put their phones on silent mode. Thus, the ringtone and vibration functions were switched off to ensure that the phones would not make any sound. All of the participants completed an intertemporal choice task (Kirby et al., 1999) using E-Prime version 2.0 (see the Supplementary material). Participants were notified that there were no correct or incorrect answers and that they should simply select the option most applicable to them. Participants were asked to choose between a smaller immediate monetary reward and a larger delayed monetary reward (e.g., $17.00 today, or $38.00 in 30 days). Options were aligned from left to right and were selected by pressing keys “F” through “J”. The computer automatically recorded participant selection and response times (RTs). Impulsive decisions were measured by the number of decisions where participants selected immediate rewards. The participants then completed an impulsive purchase intention questionnaire that contained the three consumption scenarios from study 1 (Gui et al., 2021). Next, we measured participants’ state and trait anxiety using 40 items from the State-Trait Anxiety Inventory Scale (STAI) (Spielberger et al., 1983). The participants then completed the SAS (Kwon et al., 2013). Finally, the participants provided their demographic information.

### 4.2. Data analyses

All statistical analyses were performed using SPSS (version 24.0; SPSS, Inc., Chicago, IL, USA), and the significance level was set at 0.05. We used Pearson’s correlation and a one-way analysis of variance to analyze the relationship between smartphone addiction and impulsive decision-making or impulsive purchases. A mediation analysis was performed using the PROCESS toolkit (Hayes, 2013) in SPSS. The DDM analyses used Bayesian parameter estimation implemented in the HDDM Python module to estimate the model (Wiecki et al., 2013).

#### 4.2.1. Fitting the DDM at the group level

We estimated the DDM using participants’ decisions regarding intertemporal choice for the smartphone separation and control groups. The drift rate (*v*) represents the average strength of preference for the delayed option, and it depended on the payoffs in each trial. We calculated the difference between the money (MoneyDiff) and the difference between the time delays (DelayDiff). Thus, we estimated the drift rate for each combination of MoneyDiff and DelayDiff.

#### 4.2.2. Fitting the DDM at the individual level

Individual-level DDM fit could determine whether these individual differences were a product of varying attribute weights. To assess fit quality, we simulated 500 samples from the posterior of the fitted model for each participant. We then computed summary statistics (probability of choosing the delayed reward and mean RTs associated with delayed and immediate rewards) over each simulated dataset for each participant. In the estimation, we set the drift rate (*v*) as a linear function of MoneyDiff and DelayDiff.


$$v=d_{c}+W_{m}*M\,o\,n\,e\,y\,D\,i\,f\,f+W_{d}*D\,e\,l\,a\,y\,D\,i\,f\,f$$

Thus, we estimated six parameters in total for the DDM: the relative starting point (*z*), threshold (*a*), non-decision time (*t*_0_), drift constant (*d*_*c*_), weight on MoneyDiff (*W*_*m*_), and weight on DelayDiff (*W*_*d*_).

### 4.3. Results

#### 4.3.1. Main effects of smartphone separation

**FIGURE 1:**
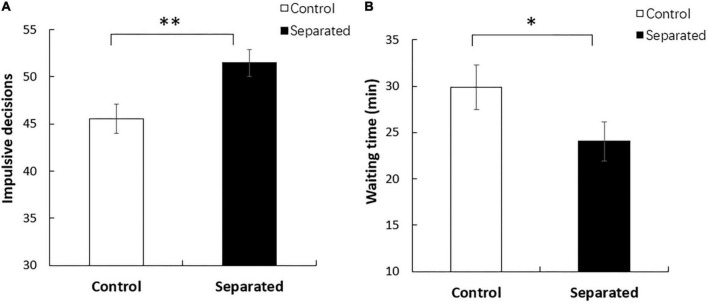
**(A)** The number of impulsive decisions made by participants in the smartphone separation condition was significantly higher than those in the control condition (*p* < 0.01). **(B)** Participants in the smartphone separation condition were less willing to wait than those in the control condition (*p* < 0.05). *p < 0.05; ^**^p < 0.01.

**FIGURE 2:**
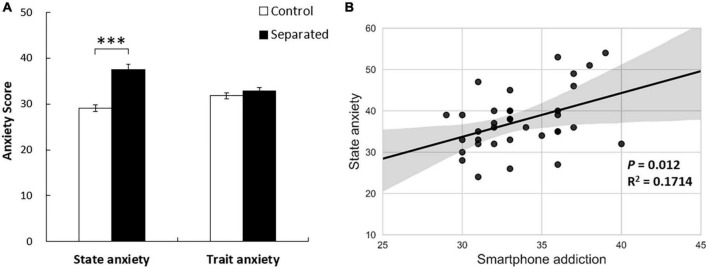
**(A)** A significant difference was observed regarding the effect of state anxiety, but no significant difference was observed with respect to trait anxiety. Error bars denote the standard error of the mean. **(B)** Smartphone addiction was positively correlated with state anxiety in the smartphone separation condition. ^***^p < 0.001.

The one-way analysis of variance indicated that the number of impulsive decisions made by participants in the smartphone separation group was significantly higher than that in the control group (*M*_*separated*_ = 51.47, SD = 8.70; *M*_*control*_ = 45.53, SD = 9.18; *F* = 3.767, *p* < 0.01, η^2^ = 0.258), with gender, age, education, job, and disposable personal income as covariates (see Figure 1A). However, no significant difference in RT was observed between the two groups. This indicates that smartphone separation only increases the tendency to select a small immediate reward. For impulsive purchases, we found that in the restaurant scenario, participants in the smartphone separation group were less willing to wait than those in the control group (*M*_separated_ = 24.06, SD = 12.59; *M*_control_ = 29.89, SD = 14.41; *F* = 2.316, *p* < 0.05, η^2^ = 0.176), with gender, age, education, job, and disposable personal income as covariates (see Figure 1B). In the movie scenario, participants in the smartphone separation group were less willing to watch the movie after the deadline (*M*_separated_ = 80.72, SD = 29.36; *M*_control_ = 87.75, SD = 17.72; *F* = 2.203, *p* = 0.054, η^2^ = 0.169) with gender, age, education, job, and disposable personal income as covariates. In the gift compensation scenario, participants in both groups showed no significant difference in the amount of compensation, with sex, age, education, job, and disposable personal income as covariates. Moreover, regarding the degree of smartphone addiction, no significant difference was observed between the two groups (*M*_separated_ = 33.58, SD = 2.83; *M*_control_ = 34.86, SD = 2.75; *F* = 3.774, *p* > 0.05, η^2^ = 0.051). This revealed that the results were affected by smartphone separation but not smartphone addiction. Specifically, the state anxiety was higher in the separation group than in the control group (*M*_separated_ = 37.50, SD = 7.25; *M*_control_ = 29.11, SD = 4.23; *p* < 0.001, *F* = 35.949, η^2^ = 0.339). However, trait anxiety did not differ significantly (*M*_separated_ = 32.83, SD = 4.35; *M*_control_ = 31.78, SD = 3.78; *p* > 0.05, *F* = 1.207, η^2^ = 0.017) (Figure 2A). These findings indicate that smartphone separation leads to higher state anxiety without affecting the trait anxiety of the participants.

#### 4.3.2. Correlations of the smartphone separation and control groups

**FIGURE 3:**
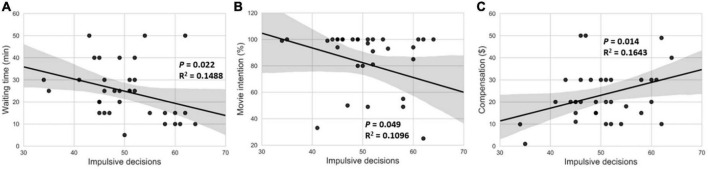
Correlation between impulsive decision-making and impulsive purchase intention in the smartphone separation condition. Impulsive decisions **(A)** significantly negatively correlated with the amount of time they were willing to wait for a restaurant, **(B)** negatively correlated with the intention of watching a movie after a critical deadline, and **(C)** positively correlated with the expected value of a compensation reward.

In the control group, there was no significant correlation between intertemporal choice and impulsive purchases in the various consumption scenarios (*p* > 0.05). Additionally, no significant correlation was observed between smartphone addiction and impulsive purchases (*p* > 0.05) or state anxiety (*p* > 0.05). The Cronbach’s alpha of SAS in study 3 was 0.808. In the smartphone separation group, a significant positive correlation was observed between smartphone addiction and state anxiety (*r* = 0.414, *p* = 0.012). This indicated that participants with higher smartphone addiction exhibited higher state anxiety (see Figure 2B). Additionally, we found that intertemporal choice was significantly negatively correlated with the time participants were willing to wait for a restaurant (*r* = 0.381, *p* = 0.022) (Figure 3A), and the intention to see a film after the deadline of a critical task (*r* = 0.331, *p* = 0.049) (Figure 3B). However, it was positively correlated with the expected value of compensation gifts (*r* = 0.405, *p* = 0.014) (Figure 3C). Participants with a greater propensity for impulsive decision making appeared to be more impulsive in their daily consumption behaviors. Therefore, intertemporal choice can significantly predict impulsive purchase. Additionally, smartphone addiction was positively (but not significantly) correlated with intertemporal choice (*p* = 0.066) and negatively correlated with the willingness to watch a movie after a critical deadline (*r* = 0.412, *p* = 0.013). However, no significant correlations were observed for the other consumption scenarios.

#### 4.3.3. Results of DDM

**FIGURE 4:**
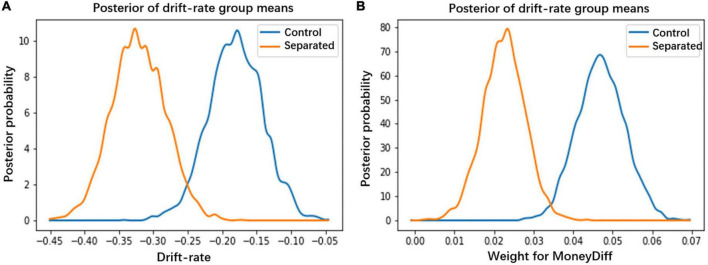
**(A)** The drift rate (*v*) in the smartphone separation condition was significantly lower than those in the control condition (*p* < 0.05). **(B)** Participants in the smartphone separation condition represented lower decision weight on MoneyDiff than those in the control condition.

We calculated the drift rate and the decision weight on MoneyDiff and DelayDiff using DDM. The results showed that the participants in the two groups had different drift rates in the dynamic accumulation of evidence during the decision-making process. The participants in the separated group exhibited lower drift rates (*p* < 0.05; Figure 4A). Furthermore, to examine how reward and time information contributed to the accumulation process, we calculated the decision weights for MoneyDiff and DelayDiff for each group. The one-way analysis of variance indicated that the decision weight on MoneyDiff was significantly lower in the smartphone separation group than in the control group (*M*_separated_ = 0.023, SD = 0.026; *M*_control_ = 0.047, SD = 0.029; *F* = 14.106, *p* < 0.001) (Figure 4B). However, regarding the decision weight on DelayDiff, no significant difference was observed between the two groups (*M*_separated_ = −0.010, SD = 0.002; *M*_control_ = −0.009, SD = 0.002; *F* = 2.046, *p* > 0.05). These results indicate that participants in the separation group had less patience than those in the control group. Thus, smartphone separation reduces individuals’ patience by lowering their decision weight on rewards.

#### 4.3.4. Correlations between anxiety and decision weights

**FIGURE 5:**
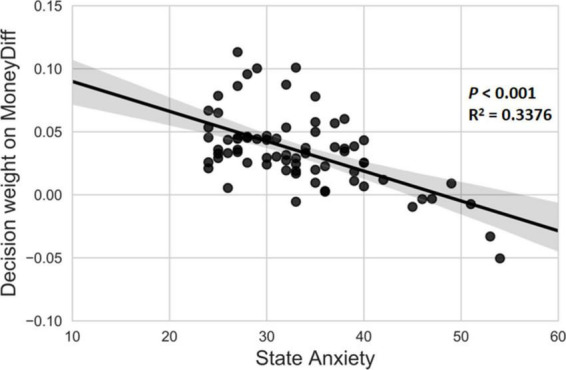
Decision weight on MoneyDiff was significantly negatively correlated with state anxiety.

No significant correlation was observed between the degree of trait anxiety and decision weight on either MoneyDiff (*p* > 0.05) or DelayDiff (*p* > 0.05). This suggests that trait anxiety does not correlate with the dynamic decision process. We noted a significant negative correlation between state anxiety and decision weight on MoneyDiff (*r* = −0.581, *p* < 0.001) (Figure 5). This suggests that participants with higher state anxiety placed less weight on MoneyDiff and were more impulsive. However, no significant correlation was found between state anxiety and decision weight on the DelayDiff (*p* > 0.05).

#### 4.3.5. Mediation analysis

**FIGURE 6:**
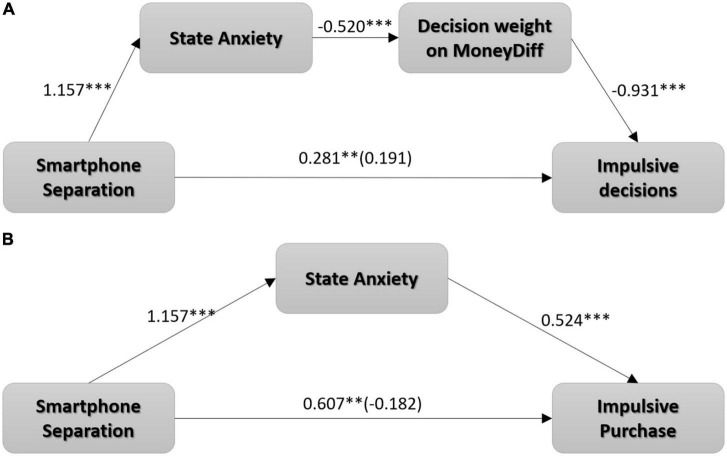
**(A)** A dual mediation model for the serial indirect effect of smartphone separation on impulsive decisions through state anxiety and decision weight on MoneyDiff. **(B)** State anxiety mediated the effects of smartphone separation on impulsive purchase in the scenario of waiting for a meal in a restaurant. ^**^p < 0.01; ^***^p < 0.001.

We standardized all variables and conducted a mediation analysis with smartphone separation (0 = control, 1 = separated) as the independent variable. The standardized number of selected immediate rewards was the dependent variable, while standardized state anxiety and standardized decision weight on MoneyDiff served as mediators. PROCESS Model 6 with 5,000 bootstrap samples was used. The results revealed significant dual mediation for the indirect effect of smartphone separation on impulsive decision-making through state anxiety and decision weight on MoneyDiff (indirect effect = 0.281, SE = 0.080, 95% CI [0.139, 0.452]) (Figure 6A). The results indicate that smartphone separation leads to higher state anxiety, thereby reducing the decision weight on MoneyDiff. Thus, participants chose a more immediate reward.

Furthermore, we standardized the reverse score of the time participants were willing to wait in the restaurant scenario as an impulsive purchase intention. With impulsive purchase intention as the dependent variable, PROCESS Model 4 with 5,000 bootstrap samples was conducted. The findings were significant for the indirect effect of smartphone separation on impulsive purchase through state anxiety (indirect effect = 0.607, SE = 0.183, 95% CI [0.295, 1.020]) (Figure 6B). This finding suggests that smartphone separation leads to higher levels of state anxiety. Thus, it led to a stronger tendency to make impulsive decisions in the intertemporal choice task and exhibit higher impulsive purchase intentions.

### 4.4. Discussion

Study 3 considered smartphone separation in order to explore its effect of smartphone separation on impulsive behaviors. First, we found that smartphone separation increased impulsive decision-making and purchases, which is consistent with hypothesis 2. Moreover, smartphone separation significantly increased the participants’ state anxiety. However, no significant effect was observed for trait anxiety, indicating that state anxiety induced by smartphone separation plays a key role. Second, in the dynamic decision-making process of intertemporal choice, individuals in the separation group exhibited lower drift rates and decision weights on MoneyDiff than those in the control group. This indicates that smartphone separation reduced individuals’ patience, supporting hypothesis 4a. However, we did not observe a significant correlation between state anxiety and decision weight on DelayDiff. Third, state anxiety mediated the effect of smartphone separation on impulsive behaviors. In particular, dual mediation analyses revealed that smartphone separation exerted significant indirect effects on impulsive decisions *via* state anxiety and decision weight on rewards. Thus, hypothesis 3 was supported. These results reveal that anxiety affected the dynamic choice process of impulsive decision-making and purchases. This incorporates anxiety and DDM, revealing a close association between individuals’ emotional states and the process of evidence accumulation.

Additionally, we found no significant correlation between smartphone addiction and impulsive behaviors in studies 1 and 2. However, for the smartphone separation group in study 3, results revealed that smartphone addiction increased individuals’ state anxiety, leading to lower patience. This indirectly increased impulsive decision-making and purchase intentions. Moreover, regardless of the degree of smartphone addiction, smartphone separation had a significant effect on impulsive decision-making and purchase intentions. This is consistent with previous research, which found that smartphone separation weakened executive function (Hartanto and Yang, 2016). Finally, we confirmed that impulsive decision-making was significantly correlated with impulsive purchase intentions among the three consumption scenarios in the case of smartphone separation.

However, it remained unclear why smartphone addiction would increase anxiety. Therefore, in study 4, we aimed to explore the underlying mechanisms of smartphone addiction and anxiety.

## 5. Study 4

In study 4, we investigated why smartphone addiction led to increased anxiety in the smartphone separation condition. Prior research has shown that FoMO (Przybylski et al., 2013), social threat (Tams et al., 2018), and extended-self (Hanley et al., 2018) may mediate the effects of smartphone addiction and other variables such as stress and executive functions. Thus, we tested whether these factors played a role in the relationship between smartphone addiction and anxiety.

### 5.1. Method

A total of 113 healthy volunteers participated in this study (65 females and 48 males), and their ages ranged from 28 to 40 years (the mean age was 25.1 years). We conducted the study online for 3 days. The sample size was determined using G*Power 3 (Faul et al., 2007), in which the statistical power was 0.9 for the one-way analysis of variance and linear multiple regression. The sample consisted of students, grassroots staff, technical staff, junior and senior managers, and other professionals. All participants provided written informed consent prior to the experiment.

We created an online survey using WJX^2^ to measure all variables in the study. The benefits of using this platform are described in detail in study 2. The participants were asked to imagine themselves in an emergency situation in which they could not use their smartphones. We then measured smartphone addiction and state anxiety using the same methods as in study 3. Next, we measured the participants’ extended-self domains, FoMO, and perceived social threat.

The extended-self domain was assessed using a single-item pictorial measure. This measurement style has been used previously to examine self-extension in broader contexts (Shvil et al., 2013; Hanley et al., 2018). Participants were presented with a set of seven Venn diagrams in which two circles overlapped to varying degrees. The degree of overlap increased uniformly, yielding a linear, seven-point scale. Participants were told that one circle represented their self, and another circle represented their smartphone. Circles that were entirely separate represented the self being completely independent from the smartphone, whereas completely overlapping circles represented the self being fully dependent on the smartphone. Participants were prompted to choose the diagram that was most applicable to themselves. Participants were then asked to complete the FoMO scale (Przybylski et al., 2013) which comprised of items such as “It bothers me when I miss an opportunity to meet up with friends.” Responses were rated on a 5-point Likert-scale, with answers ranging from 1 (“Not at all true of me”) to 5 (“Extremely true of me”). Social threat was measured using a Likert scale, adapted from Tams et al. (2018). Finally, the participants provided their demographic information.

### 5.2. Data analyses

All statistical analyses were performed using SPSS (version 24.0; SPSS, Inc., Chicago, IL, USA), and the significance level was set at 0.05. We used Pearson’s correlation and a one-way analysis of variance to analyze the relationship between smartphone addiction and impulsive decision-making or impulsive purchases. A mediation analysis was performed using the PROCESS toolkit (Hayes, 2013) in SPSS.

### 5.3. Results

**FIGURE 7:**
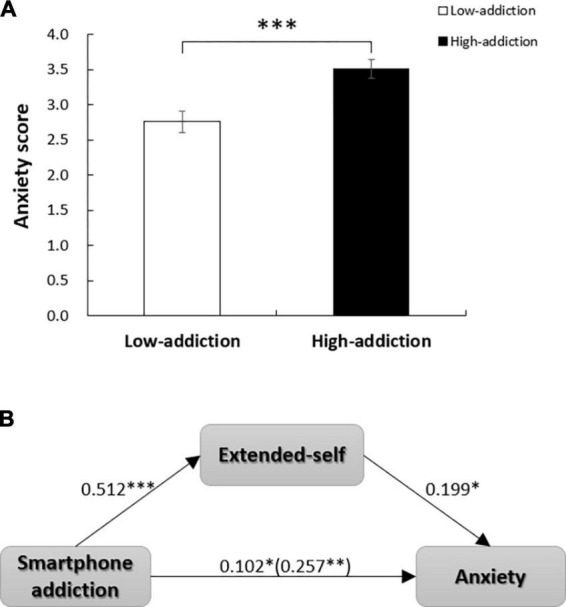
**(A)** Participants in the high-addiction group represented higher anxiety than those in the low-addiction group (*p* < 0.001). **(B)** Extended-self mediated the effects of smartphone addiction on anxiety. *p < 0.05; ^**^p < 0.01; ^***^p < 0.001.

Participants were divided into two groups based on the median score (*M* = 33) of smartphone addiction. A one-way analysis of variance indicated that participants in the high addiction group exhibited higher anxiety than those in the low addiction group (*M*_high–addiction_ = 3.51, SD = 1.01; *M*_low–addiction_ = 2.76, SD = 1.10; *p* < 0.001, *F* = 14.327, η^2^ = 0.114) (Figure 7A). The Cronbach’s alpha of SAS in study 4 was 0.779. This finding suggests that smartphone addiction significantly increases anxiety among individuals. Furthermore, participants in the high addiction group had a closer extended-self domain on their smartphones than those in the low addiction group (*M*_high–addiction_ = 4.86, SD = 0.99; *M*_low–addiction_ = 3.83, SD = 1.11; *p* < 0.001, *F* = 27.179, η^2^ = 0.197). The findings were similar for FoMO (*M*_high–addiction_ = 3.17, SD = 0.57; *M*_low–addiction_ = 2.71, SD = 0.55; *p* < 0.001, *F* = 18.941, η^2^ = 0.146) and social threat (*M*_high–addiction_ = 3.19, SD = 0.73; *M*_low–addiction_ = 2.80, SD = 0.85; *p* < 0.01, *F* = 7.059, η^2^ = 0.060). The Cronbach’s alpha of the FoMO scale was 0.718, and that of the social threat scale was 0.840. The results showed that higher smartphone addiction was related to a higher extended-self domain on the smartphone, FoMO, and social threat.

We then standardized all variables and conducted a mediation analysis. Smartphone addiction was the independent variable, anxiety was the dependent variable, and extended-self, FoMO, and social threat served as mediators. The PROCESS Model 4 with 5,000 bootstrap samples was used. The results were significant for the indirect effect of smartphone addiction on anxiety through the extended-self (indirect effect = 0.102, SE = 0.050, 95% CI [0.015, 0.209]) (Figure 7B). However, we observed no significant mediation effect for FoMO (indirect effect = 0.052, SE = 0.067, 95% CI [−0.090, 0.179]) or social threat (indirect effect = 0.032, SE = 0.038, 95% CI [−0.032, 0.125]).

### 5.4. Discussion

Study 4 examined the mediating role of these three factors in the relationship between smartphone addiction and anxiety. We found that the extended-self was a mediating factor, while FoMO and social threat were not significant. The results of study 4 revealed that smartphone addiction increased individuals’ anxiety by increasing their extended-self domain on smartphones; thus, hypothesis 5c was supported.

## 6. General discussion

Our findings provide valuable theoretical and practical contributions. First, the present research demonstrates that the connection between smartphone addiction and impulsive behavior is weak. It is inconsistent to studies that has found that behavioral addiction reduces self-control and impairs decision-making abilities, leading to a preference for immediate rewards in intertemporal choice (Li et al., 2016). Our results showed that smartphone addiction did not predict impulsive behavior unless the participants were separated from their smartphones. This may be because the degree of smartphone addiction was not high in all participants. Tang et al. (2017) divided participants into three groups according to their degree of smartphone addiction, and discovered that participants with medium and high levels of addiction exhibited greater impulsive intentions in intertemporal choice tasks. However, no significant difference was noted between the participants with high and medium levels of addiction. This suggests that the probability of selecting an immediate reward in the intertemporal choice task does not increase after smartphone addiction reaches a certain point (Tang et al., 2017). The weak correlation between smartphone addiction and intertemporal choice may be because smartphone addiction scores ranged from 11 to 55 on our scale, with many participants having a low addiction score.

However, smartphone separation increased participants’ impulsive decisions and purchase intentions. Moreover, state anxiety, but not trait anxiety, mediated this effect. The state anxiety induced by smartphone separation was correlated with smartphone addiction. This result indicates that smartphone separation impairs self-control. This is consistent with previous studies that found that smartphone separation triggers severe anxiety (Hartanto and Yang, 2016) and affects executive function and higher-order cognitive processes such as mental shifting (Hartanto and Yang, 2016), effortful reasoning (Barr et al., 2015), and working memory capacity (Ward et al., 2017).

Second, our research shows how emotional states change the trade-off between fundamental components in dynamic decision-making processes through computational modeling. We found that smartphone separation significantly increased participants’ state anxiety but not trait anxiety, suggesting that state anxiety induced by smartphone separation plays a key role in the decision-making process. Previous research has shown that anxiety-related stress levels can be predicted using the combined features extracted from smartphone log data (Fukazawa et al., 2019). Furthermore, state anxiety has been associated with individuals’ behavior and decision-making functions. For example, anxiety hampers the retrieval of specific autobiographical memories (Hallford et al., 2019). Additionally, smokers with high levels of anxiety may be at risk of ongoing smoking. That is, smokers with higher anxiety may have increased difficulty in resisting impulses to smoke (Watson et al., 2018). Our findings showed no significant differences in trait anxiety between the experimental and control groups. This indicated that the decision-making process was not affected by variations in individual characteristics but by the manipulation of smartphone separation.

Furthermore, through multi-attribute DDM, we demonstrate that smartphone separation reduces the decision weights on reward, thus increasing impulsive decisions. This finding is consistent with those reported by Amasino et al. (2019). Their research found that people who make more patient choices tend to directly compare monetary rewards and ignore delay-time information. The correlation between state anxiety and decision weight on reward and the effects of smartphone separation on impulsive decisions *via* state anxiety and decision weight on reward demonstrate the underlying cognitive mechanism of impulsive decision-making. The decision weight during the evidence accumulation process represents the integration of the two fundamental components of reward and delay, and the formation of the subjective value of intertemporal choice.

Therefore, smartphone separation induces state anxiety, and the state anxiety changes the trade-off between reward and delay. This decreases the individual’s consideration of the reward, which influences the subjective value integration process and leads to impulsive decision-making. Recent research has shown that the dynamic decision-making process is related to the amplification of benefit-versus-cost information attended to early in the decision-making process (Westbrook et al., 2020). And, our results reveal that decisions to collect immediate rewards reflect a reduction in reward-versus-delay information accumulation in the dynamic decision-making process. In addition, the reduction effect on rewards was strengthened by higher smartphone addiction.

Third, we examine the underlying psychological mechanisms of anxiety caused by smartphone separation on impulsive behaviors. Our results revealed that the extended-self domain of smartphones mediated the relationship between anxiety and smartphone addiction. The self or personal identity is considered to be an individual choice (Berger and Heath, 2007), and variety in a self-expressive assortment had detrimental effects on decision-making in many domains (e.g., intertemporal choice) (Rifkin and Etkin, 2019). Mobile technology has become an important component of personal identity. In the case of smartphone separation, individuals feel a lack of self, which increases their state of anxiety. FoMO and social threats are also important factors in decision-making, which are related to social factors. FoMO has been shown to mediate the relationship between anxiety and severity of problematic smartphone use (Elhai et al., 2019). However, no significant mediation effect was observed between problematic smartphone use and anxiety (Elhai et al., 2016). Moreover, previous research has shown that social threat mediates the relationship between smartphone separation and stress. However, anxiety differs from stress, especially in processing threatening stimuli (Mogg et al., 1990). Overall, smartphone addiction indirectly affected impulsive behavior when the extended-self domain on smartphones increased, thus enhancing anxiety in the case of smartphone separation.

Last but not least, our research indicates that smartphone separation has a negligible effect on preventing impulsive behavior, and how to decrease the anxiety caused by smartphone addiction and separation. Abrupt smartphone separation may lead to withdrawal effects, which may arouse state anxiety and lead to impulsive decision-making and purchases. Overall, smartphone separation may not produce desirable effects for individuals seeking to cure smartphone addiction or increase their work efficiency. Our results suggest that decreasing state anxiety triggered by smartphone separation and smartphone addiction is crucial for preventing impulsive behavior caused by smartphone separation. In the workplace, employees attend meetings or programs without using smartphones. For example, employees are required to leave their communication devices, especially smartphones, outside the meeting rooms. For managers who want to improve staff efficiency, impulsive intentions and anxiety caused by smartphone separation may influence work quality. Thus, it is essential to highlight that smartphones are tools and to reduce the extension of the self on smartphones. Furthermore, increasing the extent of work-related tasks and meetings may decrease employees’ anxiety, thus positively influencing self-control and working efficiency.

There are several limitations in our studies. First, this study focused on young adults. Therefore, these results may not be generalizable to other populations such as children or older adults. Older adults often have more leisure time after retirement and less working pressure. Moreover, children still develop both physically and psychologically. Thus, the participants in these two groups were more prone to smartphone addiction. Cho and Lee (2017) reported that smartphone addiction significantly affects children’s problem-solving abilities and emotional intelligence (Cho and Lee, 2017). Older adults also exhibit a predilection for engaging in alcohol and Internet addiction behaviors (Crome et al., 2012). Therefore, further research is required to elucidate the effects of smartphone addiction and impulsive behaviors in other age groups. Second, we used questionnaires and manipulated experiments to explore the relationships between smartphone separation, smartphone addiction, and impulsive behaviors. However, few studies have addressed the attention and neural mechanisms involved in impulsive purchases. Future research should employ various tools to explore this topic, such as functional magnetic resonance imaging (fMRI), electroencephalograms, or event-related potentials (EEG/ERP). Moreover, consumers are affected by various environmental factors (Isikman et al., 2016) when shopping, watching movies, playing games, and attending concerts. Because these factors are difficult to investigate in laboratory environments, future research may utilize field studies, such as experiments in restaurants and cinemas.

## 7. Conclusion

Our findings make important theoretical and practical contributions to the literature. First, our findings advance the current understanding of impulsive decision-making and purchase. We found that smartphone addiction was not strongly correlated with participants’ impulsive behaviors, unless they were separated using a smartphone. Smartphone separation significantly increased impulsive decision making and purchase. Second, we contribute to the literature on cognitive processes of decision making by exploring the dynamic decision-making process using the multi-attribute drift decision model, and found that smartphone separation reduced the drift rate and changed the trade-off between reward and delay, and the dynamic mechanism of intertemporal choice. Third, we advance research on how emotional states change decision-making processes. Our results revealed that anxiety induced by smartphone separation changed the dynamic choice processing of impulsive decision-making, and that smartphone separation increased the state anxiety of individuals, but had no significant effect on trait anxiety. In particular, state anxiety directly influences the dynamic decision process by reducing the decision weight on the reward, which represents the fundamental components of decision-making. Previous research has rarely associated emotional state and dynamic decision process; however, our investigation into individuals’ emotional state and fundamental components of decision revealed the psychological mechanism of the dynamic decision-making process. Finally, smartphone addiction led to higher state anxiety, indirectly increasing impulsive behaviors. Specifically, extended-self theory may explain the underlying psychological mechanism of this effect.

## Data availability statement

The raw data supporting the conclusions of this article will be made available by the authors, without undue reservation.

## Ethics statement

The studies involving human participants were reviewed and approved by the Shenzhen University. The patients/participants provided their written informed consent to participate in this study.

## Author contributions

SL and D-YG conceived and designed the experiments and contributed to the materials and analysis tools. YD and ZZ performed the experiments. D-YG and ZZ analyzed the data. D-YG and YD wrote the manuscript. SL provided lab equipment for running the study. All authors contributed to the article and approved the submitted version.

## Footnotes

1
https://www.wjx.com/

2
https://www.wjx.cn/
