# Supplementary material for: Losing control without your smartphone: Anxiety affects the dynamic choice process of impulsive decision-making and purchase
Source: Front Neurosci. 2023 Mar 16;17:998017. doi: 10.3389/fnins.2023.998017 (PMC10060825; doi:10.3389/fnins.2023.998017)
Supplement: Supplementary file 1 [file Image_1.pdf]

Supplementary Material

1 The procedure for the intertemporal choice task.

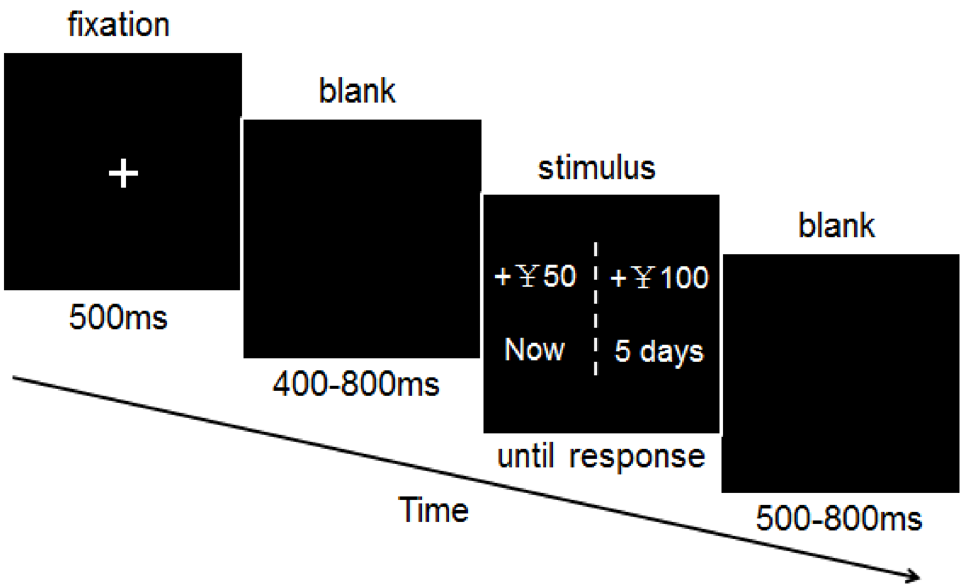

Figure S1. Time course of a single intertemporal choice trial. Each trial began with a 500-ms fixation point and was followed by the blank screen, randomized between 400 and 800 ms. A screen displaying the stimulus presentation was then shown until the participants responded. The inter-trial interval was randomized to between 500 and 800 ms.

The procedure for the intertemporal choice task is shown in Figure S1. The paradigm of intertemporal choice was adapted from previous study (Kirby et al., 1999;Li et al., 2012;Gui et al., 2016). The participants performed the experimental tasks in a small, sound-attenuated, and electrically-shielded room. The display of the stimuli and acquisition of behavioral data were conducted by E-Prime software (Version 2.0, Psychology Software Tools, Inc.) and were presented on an LCD monitor, with a 60-Hz refresh rate. During the task, the participants were positioned approximately 80 cm from the computer screen. Participants were instructed to choose between two monetary-gain alternatives; an immediate and smaller reward (IS) or a later and larger reward (LL), to be obtained at different times (e.g., $17.00 today, or $38.00 in 30 days). Participants were notified that there were no correct or incorrect answers, and that they should simply select the option most applicable to them. The two alternatives for each choice were presented on either side of the screen. The location of the immediate and delayed options was randomly assigned (left or right) on each trial and were counterbalanced across trials, and participants were instructed to press the "F" key to denote a left-side choice or the "J" key to denote a right-side choice.

Gui, D.-Y., Li, J.-Z., Li, X., and Luo, Y.-j. (2016). Temporal Dynamics of the Interaction between Reward and Time Delay during Intertemporal Choice. *Frontiers in Psychology* 7. doi: 10.3389/fpsyg.2016.01526.

Kirby, K.N., Petry, N.M., and Bickel, W.K. (1999). Heroin addicts have higher discount rates for delayed rewards than non-drug-using controls. *Journal of Experimental Psychology: General* 128**,** 78-87. doi: https://doi.org/10.1037//0096-3445.128.1.78.

Li, J., Gui, D., Feng, C., Wang, W., Du, B., Gan, T., and Luo, Y. (2012). Victims' time discounting 2.5 years after the Wenchuan earthquake: an ERP study. *PLoS One* 7**,** e40316. doi: 10.1371/journal.pone.0040316.
